# Aspirin use and knowledge in the community: a population- and health facility based survey for measuring local health system performance

**DOI:** 10.1186/1471-2261-14-16

**Published:** 2014-02-07

**Authors:** Gregory A Roth, Catherine W Gillespie, Ali A Mokdad, Danny D Shen, David W Fleming, Andy Stergachis, Christopher JL Murray, Ali H Mokdad

**Affiliations:** 1 Department of Medicine, Division of Cardiology, University of Washington, Seattle, WA, USA; 2 Institute for Health Metrics and Evaluation, University of Washington, Seattle, WA, USA; 3 Center for Translational Science, Children’s National Health System, Washington, DC, USA; 4 Department of Surgery, University of Texas Southwestern Medical Center, Dallas, TX, USA; 5 Department of Pharmacy, University of Washington, Seattle, WA, USA; 6 Public Health—Seattle & King County, Seattle, WA, USA; 7 Department of Epidemiology, University of Washington, Seattle, WA, USA; 8 Department of Global Health, University of Washington, Seattle, WA, USA

**Keywords:** Aspirin, Prevention, Coronary disease

## Abstract

**Background:**

Little is known about the relationship between cardiovascular risk, disease and actual use of aspirin in the community.

**Methods:**

The Measuring Disparities in Chronic Conditions (MDCC) study is a community and health facility-based survey designed to track disparities in the delivery of health interventions for common chronic diseases. MDCC includes a survey instrument designed to collect detailed information about aspirin use. In King County, WA between 2011 and 2012, we surveyed 4633 white, African American, or Hispanic adults (45% home address-based sample, 55% health facility sample). We examined self-reported counseling on, frequency of use and risks of aspirin for all respondents. For a subgroup free of CAD or cerebral infarction that underwent physical examination, we measured 10-year coronary heart disease risk and blood salicylate concentration.

**Results:**

Two in five respondents reported using aspirin routinely while one in five with a history of CAD or cerebral infarction and without contraindication did not report routine use of aspirin. Women with these conditions used less aspirin than men (65.0% vs. 76.5%) and reported more health problems that would make aspirin unsafe (29.4% vs. 21.2%). In a subgroup undergoing phlebotomy a third of respondents with low cardiovascular risk used aspirin routinely and only 4.6% of all aspirin users had no detectable salicylate in their blood.

**Conclusions:**

In this large urban county where health care delivery should be of high quality, there is insufficient aspirin use among those with high cardiovascular risk or disease and routine aspirin use by many at low risk. Further efforts are needed to promote shared-decision making between patients and clinicians as well as inform the public about appropriate use of routine aspirin to reduce the burden of atherosclerotic vascular disease.

## Background

Aspirin is among the least expensive and most widely available of medications yet little is known about how it is being used. Efforts to reduce the burden of cardiovascular disease, the leading cause of death in the world, have focused on improving the use of aspirin for individuals at higher risk of atherosclerotic vascular disease [1,2]. For example, in the United States, the Million Hearts initiative seeks to prevent one million heart attacks and strokes over 5 years, in part, by promoting appropriate aspirin therapy and other effective interventions. Data on aspirin use in the community is extremely limited and has generally focused on select populations of higher-risk individuals or national surveys [3-5]. Little information is available on difference in use between men and women. No methods have been developed to track the appropriateness of this widely adopted intervention at the local level.

In order to provide high-quality evidence for measuring local health system performance, we developed a novel community and health facility-based survey designed to track disparities in the delivery of interventions for common chronic diseases. We used survey questions specifically designed to investigate aspirin use and, for a subgroup undergoing physical examination, measures of cardiovascular risk and serum salicylic acid levels to explore the relationship between reported and detected aspirin use. We hypothesized that risk-treatment mismatch occurs, with unnecessary aspirin use among low-risk individuals where risk may exceed benefit as well as inadequate use for higher risk individuals and those with established disease. Based on public perception about heart disease as well as national guideline recommendations for aspirin prescription, we also hypothesized that women would receive less aspirin than men. We piloted this new chronic disease surveillance program in Seattle-King County, WA, a relatively wealthy and well-educated U.S. county where we hypothesized that health care delivery would be of high quality [6].

## Methods

### Population studied

The Measuring Disparities in Chronic Conditions Survey is a community-based survey of chronic disease designed to investigate variation in adult health and health services within a large urban county. The study was designed to address the need for high-quality health data at the local level by integrating multiple data sources including multi-mode surveys of county residents (telephone, web, mail, and in person interviews), medical and pharmacy records, administrative databases, and physical exam data. The survey included non-institutionalized white, African American, and Hispanic adults aged 18 or older living in King County, WA. With over 2 million inhabitants, King is the thirteenth most populous county in the United States and encompasses the city of Seattle as well as suburban and rural areas [6]. We sampled from both the community using home addresses and from local health facilities using billing codes for preselected common chronic conditions. Sampling from representative health facilities was designed to increase the number of respondents with chronic conditions above the prevalence typically found in community sampling.

### Analysis

In order to ascertain the use of aspirin for vascular disease, we compared MDCC survey responses separately for men and women with and without atherosclerotic cerebrovascular and coronary artery disease (CAD). Disease status was determined by the response to survey questions that asked about a history of coronary heart disease, coronary artery disease, ischemic heart disease or blocked arteries, angina, myocardial infarction, heart attack, coronary stenting, coronary artery bypass grafting, or cerebral infarction (also described as a stroke or brain attack). We defined disease as an affirmative response to any of these questions. We defined routine aspirin use as self-report of taking aspirin daily or every other day. Statistical analysis was performed using Stata 12 [7]. Approval was obtained from the institutional review board of the University of Washington.

### Sensitivity analysis using serum metabolite concentration

To validate the survey’s ability to assess disease risk and self-reported aspirin use, a subgroup of 250 consecutive survey respondents was invited to a structured examination for anthropometry, blood pressure measurement, ECG and phlebotomy. For the subgroup undergoing physical examination and without self-reported CAD or cerebral infarction, we measured cardiovascular risk factors directly, including blood pressure and cholesterol, to calculate 10-year risk of coronary heart disease events using the Framingham risk equation as described by Wilson et al. [8]. To supplement data on self-reported use of aspirin for this group, we analyzed an untimed blood sample for the aspirin metabolite salicylate using liquid chromatography-mass spectrometry according to a published method [9]. In formal pharmacokinetic studies, even at a low 25-mg dose of aspirin, serum salicylate has a half-life of 1.7 hours. Based on this, we have made the conservative assumption that salicylate levels following ingestion of 81–325 mg of aspirin should be detectable for at least 8 hours following ingestion using our high-sensitivity assay [10]. Supporting this assumption, respondents with detectable salicylate level had a median lag of 6.3 hours (range 0.25-21.75) between self-reported time of ingestion and time of phlebotomy and was undetectable in only 5 out of 96 respondents with both a self-reported time of ingestion and a measured metabolite concentration.

## Results

We found that 42% of surveyed respondents used aspirin routinely (27.8% in the community sample and 51.7% in the health facility sample). Existing disease (defined as CAD or cerebral infarction) was reported by 1117 (24.1%) of 4633 respondents (Table 1). Our study was designed to address the limitations of random population sampling by identifying more respondents with elevated risk than is found in untargeted community surveys. Supporting this hypothesis, we found only 8.1% in the community sample had disease compared with 33.6% in the health facility sample. Those with disease from both samples were older, more frequently male, and more frequently reported white/Caucasian race.

**Table 1:** Demographics for community and health facility samples

		**No history of CAD or cerebral infarction**	**%**	**History of CAD or cerebral infarction**	**%**
Community sample		1,583	91.9	139	8.1
	Age, median (IQR)	54 (42–65)		70 (59–79)	
	Male	654	41.3	80	57.6
	White, non-Hispanic	1206	76.2	114	82.0
	Black, non-Hispanic	90	5.7	8	5.8
	Hispanic	239	15.1	17	12.2
Health facility sample		1933	66.4	978	33.6
	Age, median (IQR)	62 (52–71)		68 (60–78)	
	Male	883	45.7	608	62.2
	White, non-Hispanic	1613	83.5	881	90.1
	Black, non-Hispanic	138	7.1	38	3.9
	Hispanic	96	5.0	25	2.6
Total		3516	75.9	1117	24.1
	Age, median (IQR)	59 (48–69)		68 (60–78)	
	Male	1537	43.7	688	61.6
	White, non-Hispanic	2,819	80.2	995	89.08
	Black, non-Hispanic	228	6.5	46	4.1
	Hispanic	335	9.5	42	3.8

Overall, 33.5% of respondents without disease reported routine aspirin use, compared with 72.1% of respondents with disease (chi-squared p < 0.001) (Table 2). These proportions were only slightly higher when we restricted the analysis to those not reporting a reason that made aspirin unsafe (39.5% without disease and 83.2% with disease, not shown). A discussion with a health provider on the risks and benefits of aspirin to prevent heart attack and stroke was reported by 47.9% of those without disease and 81.7% of those with disease (chi-squared p < 0.001). A health problem that made aspirin or NSAIDs unsafe was reported by 15.7% of those without disease and 24.4% of those with disease (chi-squared p < 0.001).

**Table 2:** Aspirin knowledge and use by sex

	**Male**				**Female**				**Total**			
	**No disease (N)**	**%**	**disease (N)**	**%**	**No disease (N)**	**%**	**Disease (N)**	**%**	**No disease (N)**	**%**	**Disease (N)**	**%**
**Frequency of aspirin use**												
Never	562	36.6	110	16.0	938	47.5	119	27.7	1,500	42.7	229	20.5
Less than once a week	182	11.9	15	2.2	219	11.1	16	3.7	401	11.4	31	2.8
At least once a week	42	2.7	4	0.6	60	3.0	5	1.2	102	2.9	9	0.8
Every other day	30	2.0	15	2.2	30	1.5	3	0.7	60	1.7	18	1.6
Every day	566	36.9	511	74.3	552	27.9	276	64.3	1,119	31.8	787	70.5
Don’t know or declined to respond	23	1.5	33	4.8	20	1.0	10	2.3	333	9.6	4	3.9
**Risks and benefits of aspirin to prevent heart attack or stroke ever discussed with you**												
No	708	46.1	115	16.7	1114	56.4	89	20.8	1823	51.9	204	18.3
Yes	825	53.7	573	83.3	859	43.5	339	79.0	1685	47.9	912	81.7
Don’t know or declined to respond	4	0.3	0	0	4	0.2	1	0.2	8	0.2	1	0.01
**Health problem that make aspirin or NSAIDs unsafe**												
No	1205	78.4	508	73.8	1459	73.8	294	68.5	2665	75.8	802	71.8
Yes	195	12.7	146	21.2	356	18.0	126	29.4	551	15.7	272	24.4
Don’t know or declined to respond	137	8.9	34	4.9	162	8.2	9	2.1	300	8.5	43	3.9

Use and knowledge of aspirin was associated with sex of the respondent. Significantly more men than women reported use both among those with and without disease (76.5% vs. 65.0% and 38.9% vs. 29.4% respectively, all p < .05) (Table 2). Men were more likely than women to report a discussion of risks and benefits among those without disease (53.7% vs. 43.5%) but not among those with disease. Reporting a health problem that made aspirin or NSAIDs unsafe was more common for women than men among those with and without disease (29.4% vs. 21.2% and 18.0% vs. 12.7%, respectively).

Reasons and risks for aspirin use were also explored using survey questions designed to capture the main indication for treatment and its safety. Among those reporting routine aspirin use, the most common reason for using aspirin was for the purpose of lowering the chance of heart attack or stroke, cited by 697 of 1178 (59.2%) without disease compared with 495 of 802 (61.7%) with disease. Relief of pain was infrequently cited as the main reason for routine aspirin use (59 of 1178 (5.0%) among those without disease and 16 of 802 (2.0%) with disease). When respondents were asked to identify reasons that aspirin or non-steroidal anti-inflammatory medications were unsafe for them (and allowed to select multiple answers), those without disease were most likely to report either a stomach or gastrointestinal condition (139 of 576 (24.1%)) or use of another blood thinning medication (131 of 576 (22.7%)). Among respondents with disease, the most common reasons was most likely to be use of another blood thinning medication (123 of 288 (42.7%)). Allergy (34 of 576 (5.9%) with disease and 12 of 288 (4.2%) without disease) and history of bleeding events (27 of 288 (5.9%) with disease and 56 of 576 (9.7%) without disease) were much less commonly reported reasons for avoiding the use of aspirin or non-steroidal anti-inflammatory medications. Of note, routine aspirin use occurred in both groups even when there was report of a health problem that made the use of aspirin or NSAIDS unsafe (used by 119 of 551 (21.6%) without disease, despite contraindications, and by 134 of 272 (49.3%) with disease, despite contraindications).

Aspirin dose was similar among those with and without disease even though evidence for higher-dose aspirin is limited. A dose between 80–100 mg was reported by 893 of 1337 (66.8%) with and 516 of 783 (65.9%) without disease while a dose between 300–350 mg was reported by 246 of 1337 (18.4%) with and 207 of 783 (26.4%) without disease. No significant difference in dose was seen by sex.

### Sensitivity analysis

To better understand clinical risk among those who are disease-free, we calculated the 10-year CHD risk (by Framingham equation) for the subgroup that underwent physical examination with phlebotomy. Demographics, cardiovascular risk and aspirin use for this group are shown in Table 3. Routine aspirin use was similar among individuals with both high and low CHD risk (11 of 24 (45.8%) for those with ≥10% risk vs. 55 of 160 (34.4%) for those with <10% risk, chi squared p = 0.3). These proportions did not differ significantly when we restricted our analysis to those not reporting a health condition that made aspirin unsafe. Furthermore, the proportion reporting a discussion with a health provider on the risks and benefits of aspirin or a condition that made aspirin unsafe did not differ significantly by cardiovascular risk and was similar to rates seen in the entire study population.

**Table 3:** Cardiovascular risk factors and aspirin use among examination subgroup free of disease

	**N = 184**
Age, median (IQR)	59.5 (51–67.5)
Male, n (%)	78 (42.4%)
White, non-hispanic, n (%)	161 (87.5%)
Black, non-hispanic, n (%)	8 (4.4%)
Hispanic, n (%)	15 (8.2%)
Community sample, n (%)	137 (74.5%)
Health facility sample, n (%)	47 (25.5%)
Weight, kg, median (IQR)	79.8 (66.7-92.1)
BMI, kg m2, median (IQR)	26.4 (23.6-30.7)
SBP, mmHg, median (IQR)	118 (111–123)
DBP, mmHg, median (IQR)	68 (61–75)
LDL, median (IQR)	105 (81–129)
HDL, median (IQR)	62 (47–74)
Triglycerides, median (IQR)	117 (84–171)
Current or recent (<1 yr) tobacco smoker, n (%)	16 (8.7%)
Told you have diabetes by health professional, n (%)	27 (14.8%)
Hgb A1C >6.5% or using an oral hypoglycemic medication, n (%)	24 (13.1%)
Ten-year CHD Risk <10%, n (%)	160 (87.0%)
Ten-year CHD Risk ≥10%, n (%)	24 (13.1%)
Routine aspirin use	66 (35.9%)
Risks and benefits of aspirin to prevent heart attack or stroke	
ever discussed with you	92 (50.0%)
Health problem that make aspirin or NSAIDs unsafe	21 (11.4%)

**Figure 1:**
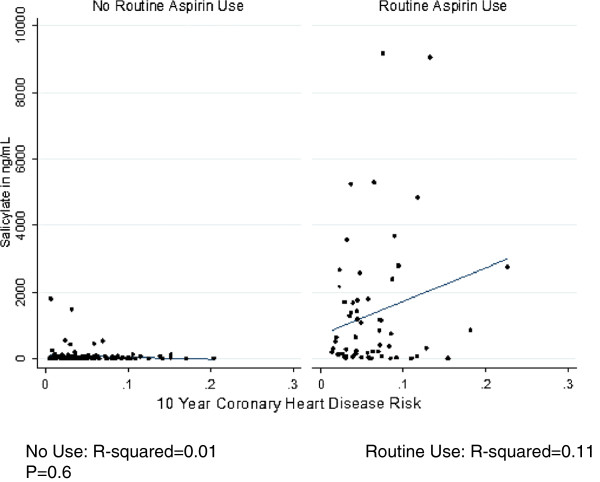
Serum salicylic acid concentration among respondents with and without routine aspirin use according to coronary heart disease risk.

Serum salicylate had a very wide range with a median detected concentration of 217 ng/ml and interquartile range 44–1415 ng/ml. Serum salicylate was not detected in 5 of the 101 (4.6%) respondents reporting routine aspirin use but was detected in 59 of the 112 (52.7%) respondents reporting no routine aspirin use (Table 4). Low concentrations of serum salicylate below 100 ng/ml have been found among many healthy subjects not using aspirin [11,12]. To address this, we identified a threshold concentration based on the receiver-operating curve for salicylate when self-reported use of routine aspirin was the gold standard. Salicylate concentration >125 ng/ml correctly classified 83% of self-reported aspirin use (74% sensitive and 91% specific for routine use). Despite the suggestion of a positive correlation, salicylate concentration was not significantly associated with calculated coronary heart disease risk in this population (p = 0.6) (Figure 1).

**Table 4:** Self-reported aspirin use and presence of serum salicylate

	**Routine aspirin use**	**No routine aspirin use**
Any detectable salicylate	96	59
No detectable salicylate	5	53

## Discussion

In this community-based survey, we found that over 40% of respondents used aspirin routinely. However use was not routine for many most likely to receive a benefit from aspirin therapy. For example, one in five with a history of CAD or cerebral infarction and no contraindication to aspirin did not use routine aspirin. Furthermore, half of subjects without disease and 18% with disease said they had never been told the risks and benefits of aspirin. Women with CAD or cerebral infarction were less likely than men to use aspirin routinely and more likely to report a health problem that would make aspirin unsafe.

Among a smaller subgroup undergoing precise measurement of cardiovascular risk, more than half at high risk for developing CAD did not use routine aspirin. A third of respondents with low cardiovascular risk used aspirin routinely. Of those who did report routine aspirin use, only 5% had no detectable salicylate in their blood, a metabolite that should have been present among daily users of aspirin. This suggests that self-report as obtained in this survey is a reasonable though imperfect measure of actual aspirin use. The reason for disagreement between self-report and serum level is unclear and suggests a difference in salicylate clearance or the accuracy of self-report for a small subset of respondents.

Prior data has suggested that aspirin is used routinely by only 30-60% of individuals who self-report a history of heart disease [3]. Our study found a slightly higher rate of reported use among those with disease. One study has reported a population-based estimate of aspirin use and found one in five of all adults took aspirin routinely [5]. Similarly, the REGARDS study found 31.5% of individuals free of CHD and stroke reporting use of aspirin [13]. We found over a quarter of adults used routine aspirin in our community sample but, after considering objectively measured cardiovascular risk, use was closer to 35% among individuals with low cardiovascular risk and 45% among individuals with high risk. Our results provide additional evidence that the decision to routinely use aspirin among healthy adults is not well-informed by their actual risk of cardiovascular disease.

The risk assessment and decision-making needed for appropriate use of aspirin are complex. Clinical trials have shown that aspirin has a protective effect for many people with an elevated risk for cardiovascular events [14-16]. However, a recent meta-analysis of primary prevention trials shows that nonfatal myocardial infarction, but not stroke or death, was reduced (with a number needed to treat around 150) and only when the study had a control population incidence of myocardial infarction over 8% over 10 years [17]. Based on this finding, the U.S. Preventative Health Task force has recommended that a reasonable risk threshold for considering the use of aspirin for the primary prevention of cardiovascular events is a predicted ≥10% event rate over the following 10 years [2]. This recommendation has been complicated by considerable disagreement over the precise benefits of aspirin as well as the best way to define the higher risk subpopulation most likely to benefit. Joint U.S. professional society guidelines recommend consideration of score-based cardiovascular risk while European guidelines recommend primary prevention aspirin only for individuals with hypertension [18,19].

While the role of aspirin in clinical practice continues to be debated, it remains the most common regularly taken medication in the United States. This fact reflects a broad popular acceptance of aspirin as well as its easy availability and low cost. Despite its ubiquity, the real-world use of aspirin is not well-understood. Because it is often obtained over-the-counter and without pharmacy benefits, databases and electronic health records often fail to capture aspirin use among the general population. Other efforts to study aspirin use, such as the REACH registry, included only higher-risk individuals who visited outpatient clinics [20]. Further complicating matters is the use of aspirin for a range of symptoms and diseases beyond reduction of cardiovascular risk, though our results show routine aspirin use to treat pain to be much less common than its use for prevention of vascular disease. Whether aspirin has a role in the prevention of cancers is not yet clear [21].

What is known about aspirin comes primarily from self-report during health survey interviews [3,5,22]. The reliability of self-reported use of aspirin has been questioned [23]. Our measurement of serum salicylate adds to the understanding of real-world use of aspirin derived from health surveys. An untimed measurement of serum salicylate >125 ng/ml was able to correctly classify most self-reported use of aspirin. However, medication adherence is a complex, recurring, and private behavior for which no perfect gold-standard exists. It is likely that we will continue to misclassify medication adherence in health interview surveys until biomarkers for health interventions are more widely adopted.

We note that our study population is from a single county and may not be generalizable to other regions. Similar to the U.S. as a whole, King County has a median age of 37 and is approximately 70% white race. However, income is higher (median household income of $70,567 in King County vs. $52,762 nationally) [6]. The REGARDS study found lower aspirin use among those of black race and lower income, suggesting that our results may not reflect poorer, more racially diverse counties [13]. Also, cardiovascular outcomes could not be assessed due to the cross-sectional nature of the sample, though the Framingham risk score is a well-validated prediction tool used routinely in clinical settings. We rely on self-reported history of vascular disease but use a broad range of questions phrased in lay language (for example, cerebral infarction is also described as stroke and brain attack). A larger sample size for physical examination was not possible due to funding constraints and, therefore, we cannot rule out the possibility that real differences went undetected in this subgroup. Despite these limitations, the use of anthropometry and biomarkers makes it possible to consider preventative aspirin use in relationship to actual rather than perceived or self-reported risk. Because respondents were sampled from both the community and health facilities, the entire MDCC cohort cannot be taken as a direct representation of the entire county population. Rather, the goal of this mixed design was to avoid the selection bias of prior studies performed in clinics and hospitals while still identifying an adequate number of respondents with common chronic diseases. In comparison, large population health surveys include relatively few respondents with chronic diseases. Medical and pharmacy records collected as part of the MDCC study, currently being abstracted at the time of our analysis, will provide further clinical details about this cohort.

## Conclusion

Our study, based on self-report and serum metabolite level, confirms insufficient aspirin use among those with high cardiovascular risk or disease and routine aspirin use by many despite low risk. These findings are from a large and well-educated urban county where health care delivery should be of high quality. Reasonable disagreement over the appropriate clinical use of aspirin for primary prevention should not prevent significant health gains from being achieved by improved aspirin use among individuals at high risk for coronary events. Our results highlight the need for strategies that better identify at-risk individuals and promote shared-decision making between patients and clinicians in order to decrease the burden of cardiovascular disease. In addition, further effort may be required to inform the public about risks, benefits, and appropriate use of aspirin.

## Competing interests

The authors declare that they have no competing interests.

## Authors’ contributions

GR had full access to all data in the study and takes responsibility for the integrity of the data and the accuracy of the data analysis. GR helped design the MDCC study, performed the data analysis and drafted the manuscript. CG, AAM, DF, AS, CM, and AHR designed and carried out the MDCC study and helped to draft the manuscript. DS provided LC-mass spectrometric analysis and helped to draft the manuscript. All authors read and approved the final manuscript.

## Pre-publication history

The pre-publication history for this paper can be accessed here:

http://www.biomedcentral.com/1471-2261/14/16/prepub
